# Oncogene addiction and tumor mutational burden in non‐small‐cell lung cancer: Clinical significance and limitations

**DOI:** 10.1111/1759-7714.13246

**Published:** 2019-12-04

**Authors:** Elisabeth Smolle, Katharina Leithner, Horst Olschewski

**Affiliations:** ^1^ Division of Pulmonology, Department of Internal Medicine Medical University of Graz Graz Austria

**Keywords:** Mutational burden, non‐small cell lung cancer, oncogene addiction

## Abstract

Lung cancer incidence has increased worldwide over the past decades, with non‐small cell lung cancer (NSCLC) accounting for the vast majority (85%) of lung cancer specimens. It is estimated that lung cancer causes about 1.7 million global deaths per year worldwide. Multiple trials have been carried out, with the aim of finding new effective treatment options. Lately, special focus has been placed on immune checkpoint (PD1/PD‐L1) inhibitors which impact the tumor immune microenvironment. Tumor mutational burden (TMB) has been found to predict response to immune checkpoint inhibitors. Conversely, recent studies have weakened the significance of TMB as a predictor of response to therapy and survival. In this review article, we discuss the significance of TMB, as well as possible limitations. Furthermore, we give a concise overview of mutations frequently found in NSCLC, and discuss the significance of oncogene addiction in lung cancer as an essential driver of tumorigenesis and tumor progression.

## Key points

1. Tumor mutational burden (TMB) predicts response to immune checkpoint inhibitors
2. Recent studies have shown, however, that TMB has significant limitations
3. Oncogene addicted cancers are driven by one predominant driver mutation
4. Targeted agents are used as first‐line treatment in certain types of NSCLC

## Introduction

Non‐small cell lung cancer (NSCLC) constitutes approximately 85% of lung cancer specimens. Increasing incidence rates of lung cancer have been observed in recent years worldwide, rendering this disease the second most common newly diagnosed cancer in men, and the fourth most common in women.^1^, ^2^, ^3^, ^4^ Throughout the last few decades, lung cancer has been the most fatal of all cancers,^5^ accounting for most cancer‐related deaths in both males and females, leading to 1.7 million global deaths per year.^2^ Worldwide, the age‐standardized incidence‐to‐mortality rates are 1.14/100 000 in men and 1.23/100 000 in women.^6^ It is estimated that nowadays 12.9% of all new cancer diagnoses is lung cancer.^6^ Mortality ranges between 25%–30%, and late diagnosis due to oligosymptomatic presentation is common.^7^, ^8^ Hence, five‐years survival rates range from 16%–20% on average, with around 73% in stage IA disease to 13% in stage IV disease.^9^ In the last three decades, numerous trials have been conducted across the globe, focusing on the establishment of new treatment options for NSCLC, and several promising results have emerged.^10^, ^11^ The field of prognostic biomarkers in lung cancer, especially to determine the effectiveness of various targeted treatment options, is quickly evolving.^10^ Tumoral immune microenvironment is a major component of solid cancers. Thus, in NSCLC cytotoxic T CD8+ lymphocyte density in association with the immunologic microenvironment, ie. tertiary lymphoid structures, impacts survival. Research has recently focused on targeting these tumor‐surrounding immune cells by PD1/PD‐L1 inhibitors. Immune checkpoint inhibitors have proven especially useful in advanced NSCLC so far, showing better effectiveness than second‐line chemotherapy.^12^ Pembrolizumab, for instance, has outperformed first‐line chemotherapy in NSCLC with strong positivity for PD‐L1.^13^ In recent years, more and more personalized treatment options for NSCLC have been developed as an add‐on to existing conventional chemotherapies, and thereby median overall survival has increased significantly within the past decade,^14^, ^15^, ^16^ ie. from 12.4 months for patients diagnosed in 2004–2009 to 14.8 months for patients diagnosed in 2010–2013 (*P* < 0.001), according to the recent literature.^17^

With this review of the literature we aim to give an overview on oncogene addiction and mutational burden. The association of oncogene addicted NSCLC and a high TMB with survival and response to therapy is discussed in‐depth. Nowadays it is assumed that all cancers are “oncogene addicted,” meaning that a single driver mutation is the primary cause of tumor progression and metastasis. However, the primary driver mutation is not always known. According to recent investigations, TMB also influences prognosis in patients whose tumors harbor classical driver mutations, such as *EGFR‐*mutations.^18^ For instance, it was found that *EGFR‐*mutant lung cancers featured lower overall TMB as compared to *EGFR‐*wild‐type cancers. High TMB in *EGFR‐*mutant lung cancers was linked to a significantly shorter overall survival in patients treated with EGFR‐TKIs.^18^ This finding stands in contrast to what is observed for TMB and treatment with immune checkpoint inhibitors. A meta‐analysis including the Embase, PubMed and Cochrane library databases, found that high TMB predicts better efficacy of immunotherapy in several cancer specimens.^19^ According to this large analysis of 103 078 cancer patients, high TMB correlated with better overall survival and better overall response rates to immunotherapy, independently of cancer type and TMB detection method.^19^

In addition, this article details the most frequent mutations found in NSCLC, and the respective indications for targeted therapeutics. We combine the subjects of TMB, somatic mutations within the tumor tissue, oncogene addiction and therapeutic implications. Although the role of oncogenic driver mutations in carcinogenesis is undoubted, their detection in noncancerous tissue, however, to a much lesser degree as opposed to cancer tissue raises new questions.^20^, ^21^, ^22^

Table 1 gives an overview of targeted therapeutics currently available for NSCLC treatment, with respect to the mutations found in a given cancer specimen (Table 1).

**Table 1:** Targeted treatment options for mutations frequently found in NSCLC

TYPE OF MUTATION	EPIDEMIOLOGY	CLINICAL IMPLICATIONS	TARGETED THERAPEUTICS	REFERENCES
a)				
*EGFR*	In NSCLC, *EGFR* mutations have been observed in 43%–89% of cases, globally.	*EGFR* mutations result in constitutive activation of signal transduction pathways, promoting cell proliferation and evasion of apoptosis.	EGFR‐targeting TKIs: Gefitinib (Iressa); Erlotinib (Tarceva) – can also be used in advanced‐stage patients without *EGFR* mutations if chemotherapy does not work; Afatinib (Giotrif); Osimertinib (targets T790M mutation as well; Tagrisso); Dacomitinib (Vizimpro)	11, 41, 43.
	1/4 of NSCLC harbor mutations in the *EGFR* tyrosine kinase domain, leading to an increased receptor expression in 75% of cases.			
		Recently, EGFR‐targeting agents have become standard first‐line treatment options for selected NSCLC patients with *EGFR* mutations.		
	>90% of *EGFR* tyrosine kinase domain mutations are found as short in‐frame deletions in exon 19 or as point mutations in exon 21.			
		Common side effects of all EGFR inhibitors include: skin disorders, diarrhea, moth sores, and loss of appetite	Monoclonal antibodies: Necitumumab (only used for squamous cell NSCLC; Portazza)	
	*EGFR* mutations are more common in women and people who have not smoked.			
ALK	About 5% of NSCLCs feature a rearrangement of the *ALK* gene.	ALK inhibitors are used after chemotherapy, or instead of chemotherapy in patients with *ALK* gene rearrangements.	Crizotinib (Xalkori), the first‐class ALK TKI showed superiority to platinum‐pemetrexed chemotherapy in *ALK*‐positive NSCLC. Crizotinib has shown >60% overall response rate in *ALK*‐positive NSCLC, improving PFS significantly as compared to second‐line chemotherapy. Ceritinib (Zykadia), a next‐generation ALK inhibitor, has shown durable response in NSCLC patients pretreated with another ALK TKI, and in ALK TKI naive patients. Alectinib (Alecensa) has shown systemic and central nervous system effectiveness in NSCLC Brigatinib (Alunbrig) Lorlatinib (Lorbrena).	11, 62–65.
	This change is most often seen in non‐smokers (or light smokers), and in the adenocarcinoma subtype.			
		Several ALK inhibitors are also useful when *ROS‐1* gene mutations are present.		
		Common side effects of ALK inhibitors are: nausea and vomiting, diarrhea, constipation, fatigue, changes in vision		
b)				
*ROS‐1*	ROS proto‐oncogene 1, receptor tyrosine kinase *(ROS‐1)* mutations occur in about 1%–3% of NSCLC specimens.	*ROS‐1* shares certain characteristics with the *ALK* oncogene.	Crizotinib (Xalkori) has been proven a therapeutic option in *ROS‐1* mutated NSCLC specimens.	52, 57, 69.
		Side‐effects of Crizotinib include anemia, leuko‐/neutropenia, nausea, vomiting, diarrhea, dizziness and impaired vision.	Crizotinib is FDA‐approved for patients with advanced, *ROS‐1* positive NSCLC.	
			Lorlatinib, a novel brain‐penetrant ALK and *ROS‐1* TKI, showed systemic and intracranial activity in *ALK*‐ and/or *ROS‐1* positive patients.	
		Lorlatinib (Lorviqua) may cause hyperlipidemia, headache, diarrhea, nausea, pneumonitis, joint pain, edema and fatigue.		
			Entrectinib (Ignyta) was FDA‐approved only for molecularly defined subsets of NSCLC, and may also be effective in *ROS‐1* mutated cancers.	
*BRAF*	*BRAF* mutations are observed in 4%–8% of NSCLC cases, causing downstream activation of the MAPK signaling pathway.	In *BRAF*‐mutant NSCLC specimens, treatment with selective *BRAF* inhibitors were effective in 33% of patients, improving median PFS with 5.5 months.	Dabrafenib (a newer‐generation, reversible kinase inhibitor of V600E‐mutant BRAF with a higher affinity than the wild‐type enzyme for mutant BRAF).	40, 55, 56, 66.
			Vemurafenib, Selumetinib, Binimetinib, PLX8394, RXDX‐105, LXH254+LTT462, AUY922 and Regorafenib are currently investigated in cinical trials in *BRAF*‐mutant NSCLC.	
		Nearly all patients harboring these mutations are active or former tobacco smokers. Even though V600E substitutions are the most common among *BRAF* mutations, one series also reported a 39% prevalence of G469A substitutions in lung cancer.		
		Side effects of Dabrafenib and Vemurafenib include decreased appetite, headache, cough, nausea, emesis and/or diarrhea and joint pain. Skin cancer incidence increases upon treatment with *BRAF* inhibitors.		
*RET*	In about 1%–2% of NSCLC, *RET*‐involving gene rearrangements have been reported.	No *RET*‐selective inhibitors have been developed yet. Multitarget agents featuring anti‐*RET* activity as well are available.	Anti‐*RET* multikinase inhibitors currently available are: Vandetanib (targets VEGFR, EGFR and RET); Cabozantinib (VEGFR2, MET, AXL, c‐KIT, FLT3, TIE2, RET); Lenvatinib (VEGFR1‐3, PDGFRB, c‐KIT FLT3, RET); Sunitinib (VEGFR1‐3, PDGFRB, c‐KIT, FLT3, RET); Sorafenib; Dovitinib; AD80 and Sitravatinib	53, 54, 58.
		*RET*‐rearrangement was associated with a high overall response rate and long PFS in NSCLC patients upon pemetrexed‐based chemotherapy.		
		Possible side effects of *RET*‐targeting multikinase inhibitors are: upper respiratory tract infections, urinary tract infections, decreased appetite, headache, sleep disturbances and impaired vision		
			Trials about *RET*‐inhibitors are still ongoing: Alectinib, Pontatinib and Apatinib are investigated specifically in *RET*‐rearranged NSCLC.	

### Tumor immune microenvironment as an anchor point for cancer therapy

Immunological aspects in the process of tumor formation, disease progression and metastasis have become ever more important for the development of new therapeutic options in recent years. Nonsynonymous somatic mutations in the coding region of a gene have been shown to generate presentable neoantigens, which are consecutively recognized by T‐cells with structurally divergent and antigen‐specific T‐cell receptors. When activated by antigen stimulation, T‐cells expand within the tumor tissue and execute their cytosolic immune function, controlling tumor growth. Intratumoral T‐cell repertoires are enriched with tumor antigen‐specific and clonally expanded T‐cells.^23^ TMB has recently been identified as a genetic signature associated with a favorable response to immunotherapies, especially immune checkpoint inhibitors.^24^ Higher TMB favors neoantigen‐specific T‐cell infiltration and oligoclonal expansion.^25^ Neoantigens were found to be widely heterogeneous between the investigated patients.^26^ High‐frequency T‐cell receptors were also distinct for each individual patient. Summing up this investigation, the link between the immunologic tumor microenvironment and TMB becomes evident.

TMB impacts treatment response to immune checkpoint inhibitors.^24^ Although the effectiveness of immune checkpoint inhibitors has clearly been demonstrated in the past, response rates vary greatly among individuals. Hence, TMB is a valid tool to predict whether a good response rate is expected. TMB is defined as the overall number of nonsynonymous mutations per coding areas of a tumor genome. TMB was previously determined by means of whole exome sequencing; however, due to high costs, targeted panel sequencing is currently being explored as a measuring tool for TMB. TMB has been demonstrated to correlate with the response to immunotherapy treatment according to several studies.^27^, ^28^, ^29^ It has been hypothesized that neoplasms with a higher mutational burden also express neoantigens more frequently, and usually induce a more pronounced immune response upon treatment with immune checkpoint inhibitors.^30^ The recently published CheckMate 227 trial showed a longer progression‐free survival (PFS) of NSCLC patients with tumors featuring a TMB of ≥10 mutations per megabase, independent of PD‐L1 expression.^31^ However, new data stemming from the CheckMate 227 trial which was presented at the ESMO 2019 congress, has weakened the significance of TMB as a predictor of treatment response.^32^ This data shows that TMB, whilst being a predictor of PFS upon therapy with ipilimumab/nivolumab, does not predict overall survival with the same reliability.^31^, ^32^ Similarly, the Keynote 189 study showed that TMB was not significantly associated with the efficacy of pembrolizumab plus chemotherapy, as compared to placebo plus chemotherapy in NSCLC.^33^, ^34^ Garassino *et al*. randomized 616 patients to either pembrolizumab plus chemotherapy or pembrolizumab in combination with placebo. TMB, as determined by whole‐exome sequencing, was not significantly associated with overall survival, PFS or objective response rate in this setting,^34^ as presented at the 2019 IALC World Conference in Barcelona. Also presented at this congress was data from the Keynote 021 study,^35^, ^36^ showing that TMB was not associated with objective response rates, PFS or overall survival in patients suffering from NSCLC who received pembrolizumab, carboplatin and pemetrexed, or carboplatin and pemetrexed alone. In this cohort of patients with metastatic nonsquamous NSCLC, TMB did not predict treatment efficacy, and TMB was also not significantly associated with PD‐L1 expression. Notably, in the pembrolizumab plus chemotherapy‐group, objective response rate was high in both the TMB‐low and TMB‐high group.^36^ A recent study, where microsatellite instability status was determined as another predictor for treatment response to immune checkpoint inhibitors, found that TMB does not correlate very well with microsatellite instability, or with PD‐L1 status.^37^ Moreover, the relationship between TMB, microsatellite instability and PD‐L1 seems to vary greatly among cancer subtypes, showing a distinct overlap in colorectal cancer, but rather poor concordance in endometrial, ovarian, neuroendocrine and cervical cancer.^37^

### Frequent genetic aberrations found in NSCLC: About ALK, EGFR and ROS‐1

Molecular testing for epidermal growth factor receptor *(EGFR)* mutations and anaplastic lymphoma kinase *(ALK)* rearrangements have become standard diagnostic procedures in the management of NSCLC.^38^, ^39^ DNA mutations in the *EGFR* gene, as detected by polymerase chain reaction (PCR), may occur in regions corresponding to the extracellular or the intracellular portions of the EGFR protein. In NSCLC, mutations affecting the intracellular portions of the protein have been observed in 43%–89% of cases, globally.^40^ One quarter of NSCLC harbor mutations in the EGFR tyrosine kinase domain which were associated with an increased receptor expression in 75% of cases.^41^, ^42^ Over 90% of EGFR tyrosine kinase domain mutations are found as short in‐frame deletions in exon 19 or as point mutations in exon 21; the latter resulting from a replacement of leucine by arginine at codon 858 (L858R).^43^ In Asian countries, about 30%–50% of NSCLC harbor activating mutations of the *EGFR* gene according to a recent report, rendering the respective patients suitable candidates for treatment with *EGFR*‐targeting tyrosine kinase inhibitors (TKIs).^44^, ^45^ All kinds of *EGFR* mutations result in constitutive activation of signal transduction pathways, leading to cell proliferation and evasion of apoptosis. Generally, somatic mutations in NSCLC can lead to oncogene activation by means of various mechanisms, eg. point mutations, insertions, deletions and/or gene rearrangements.^46^ In most cases, the gene alterations are mutually exclusive, with one predominant driver mutation in a given cancer.^47^, ^48^

Oncogenic fusion proteins also occur in NSCLC, such as *ALK*, and the ROS1 proto‐oncogene receptor tyrosine kinase (ROS‐1).^46^, ^49^, ^50^, ^51^ Besides these well‐known fusion proteins, other gene rearrangements that involve kinase‐encoding genes have also been described in lung cancer, namely the rearranged during transfection gene *(RET)*,^52^, ^53^ the neurotrophic receptor tyrosine kinase 1 *(NTRK1)*, the b‐raf proto‐oncogene, serine/threonine kinase *(BRAF)*,^54^, ^55^ and, as mentioned above, *EGFR*.^46^ For *ALK* and *ROS‐1* there are targeted therapeutics available which have already been well‐established in everyday clinical practice. In selected patients, these targeted agents show significant benefits, leading to tumor reduction and a prolongation in overall survival.^49^, ^50^, ^56^

### Oncogene addiction – a driver of tumorigenesis, but also a predictor of treatment response to ALK‐, EGFR‐ and ROS‐targeting agents

The term “oncogene addiction” is a hallmark of cancer, meaning that cancers are primarily driven by divergent signaling pathways of oncogenes. Usually this term refers to a given cancer being driven by one particular mutation, which leads to a cascade of other procarcinogenic events and pathways. In NSCLC particularly, oncogenic gene rearrangement frequently leads to expression of oncogenic fusion proteins by the oncogene formation by a 5′ partner creating an in‐frame gene fusion with a 3′ proto‐oncogene.^57^, ^58^, ^59^ Prominent examples are the Echinoderm microtubule‐associated protein‐like 4 *(EML4)‐ALK* fusion gene, *ROS‐1‐* and *RET‐*fusions. *ALK‐*rearrangements have been discovered recently as an important driver mutation in NSCLC, and especially in advanced‐stage NSCLC, *ALK‐*mutation status has a major impact on how patients are treated.^60^, ^61^, ^62^, ^63^, ^64^ *ALK* rearrangement is found in 3%–7% of patients suffering from stage IIIB or stage IV NSCLC, according to previous data.^65^, ^66^, ^67^ Adenocarcinomas harbor *ALK* mutations more frequently than squamous cell carcinomas. Interestingly, if only non‐smoker patients are considered, the prevalence of *ALK* rearrangement is much higher, ranging from 17% to 20%, depending on the case series.^65^, ^67^

Crizotinib is an *ALK*, *ROS‐1* and *MET* tyrosine kinase‐inhibitor that can be administered orally, and has shown >60% overall response rates in heavily pretreated patients with *ALK*‐positive NSCLC in single‐arm phase I and phase II trials.^61^, ^68^ Crizotinib led to significantly higher response rates and longer PFS as compared to conventional chemotherapy as second‐line treatment, and also compared to platinum/pemetrexed chemotherapy in untreated advanced NSCLC featuring *ALK‐*rearrangement. Hence, crizotinib has become standard‐of‐care in these subgroups of patients.^69^, ^70^, ^71^

Mutations of the *EGFR* gene constitute about 43%–89% of NSCLC cases, depending on the case series, rendering the affected patients candidates for targeted treatment with *EGFR*‐TKIs.^72^, ^73^, ^74^, ^75^, ^76^, ^77^, ^78^ Although screening for *EGFR* mutation‐status is performed routinely, little is known about possible coexisting carcinogenic mechanisms in the population of patients featuring *EGFR* mutation. Therapeutic benefits by the supplemental use of other biologicals to further improve survival and quality of life have become evident according to clinical trials, and some targeted agents are already used as first‐line treatment for selected patients with *EGFR* mutations.^72^, ^79^ Gefitinib, erlotinib and afatinib today constitute the standard of care drugs incorporated in routine clinical management of *EGFR*‐mutated lung cancers. Experimental studies have been carried out by several research groups where molecular subtyping using the latest DNA sequencing techniques was performed in NSCLC patients. As a result of these studies, it became clear that there is a high degree of molecular heterogeneity in *EGFR* nonmutated NSCLC.^80^ Beyond NSCLC harboring *EGFR‐* or other common mutations which can be targeted by biologicals, there is a certain gray zone of tumors as well (about 40% of NSCLC) which are negative for all molecular alterations that can be targeted by clinically approved drugs. Hence, conventional chemotherapy is the only option in these cases.^72^, ^81^, ^82^, ^83^

Facchinetti *et al*. have reviewed in‐depth *ROS‐1* inhibition in the context of oncogene addiction in NSCLC.^84^ *ROS‐1* has been shown to share certain characteristics with the *ALK* oncogene; however, there are also distinct disparities between the two fusion genes.^84^ When more common genetic aberrations, such as *EGFR‐*, *KRAS‐*, and *ALK‐*mutations, are absent it is mandatory to search for *ROS‐1* rearrangement. According to the literature, *ROS‐1* can be found in 1%–2% of lung adenocarcinomas. Crizotinib can have a major positive impact on *ROS1‐*mutated tumors, rendering this treatment option the major reason why *ROS‐1‐*rearrangements must not be overlooked.^84^ The authors of the above‐mentioned review article suggest careful standardization of molecular diagnostics, relying upon practical and efficient algorithms, and comprising immunohistochemistry as well as fluorescence‐in*‐*situ hybridization (FISH). By carefully outlining every *ROS‐1‐*mutated tumor, larger amounts of data will be gathered to gain even more insight into the tumor biology and molecular behavior in this specimen of oncogene‐addicted NSCLC.

The *ALK‐EML4* translocation represents another mutation that may be found in NSCLC, influencing treatment and prognosis. Noteworthy, it has been proposed that also the impact of radiotherapy is partly dependent on mutation status.^85^ Radiotherapy poses an important therapeutic option in locally advanced lung cancer. Hence, Dai and colleagues sought to investigate whether there are synergistic effects when combining radiotherapy and *ALK*‐inhibition via TAE684 in *ALK*‐positive versus wild‐ type lung cancer cells. In this cell culture experiment, three different cell lines were investigated: human NSCLC cell lines harboring wild‐type *ALK* (A549), human NSCLC cell lines featuring *ALK‐EML4* translocation (H3122), as well as murine Lewis Lung Cancer (LLC) cells. The three cell lines were irradiated with 1–4 Gy X‐Rays (320 keV) and carbon ions. Consecutively, TAE684, a potent and selective small‐molecule *ALK* inhibitor, was administered at the dose range of 0–100 nM. The authors assessed cell survival, proliferation rates, and apoptosis via caspase 3/7 expression levels. It was found that TAE684 inhibited the proliferation of H3122 cells in a dose‐dependent manner, as opposed to A549 and LLC cells that were more resistant to TAE684 treatment, and the half maximal inhibitory concentration was not reached at all tested concentrations (up to 100 nM); whereas the half maximal inhibitory concentration was reached at about 8.2 nM for the H3122 cells.^85^ Those anti‐proliferative effects of TAE684 were augmented by radiotherapy in H3122 cells, rendering these cells particularly more vulnerable to particle therapy with carbon ions (sensitizer enhancement ratio of about 1.61; *P* < 0.05). Moreover, activity of caspase 3/7 was evidently enhanced after combining both therapy options in H3122 cells. Summing up this interesting data, synergistic effects of combined TAE684 and radiotherapy in *EML4‐ALK* positive lung cancer cells are demonstrated: Not only does *ALK*‐inhibition enhance the effect of canonical photon radiotherapy, but also of particle irradiation using carbon ions.^85^

### RET‐and c‐MET proto‐oncogenes: Less frequent examples of oncogene addicted NSCLC

The *RET* proto‐oncogene encodes a certain receptor tyrosine kinase which is found in tissues that originate from the neural crest. *RET* also plays a role in the development of the kidneys and the enteric nervous system.^86^, ^87^ When gene rearrangements involve *RET*, cells acquire oncogenic properties, as has been proven in thyroid papillary carcinoma.^88^ In about 1%–2% of NSCLC, *RET*‐involving gene rearrangements have been reported, according to the recent literature. Interestingly, NSCLC gene rearrangements involving *RET* are mutually exclusive with other procarcinogenic driver mutations, eg. *ALK* or *ROS1* rearrangements.^57^, ^89^, ^90^ Multiple fusion partners with *RET* have been described: According to a global registry where patients with *RET*‐rearranged NSCLC were involved, it was reported that among 81 cases with identified *RET*‐fusion partners, the kinesin family 5B gene *(KIF5B)* was involved in 72%. This finding supported previous observations where *KIF5B* was also described as the most prevalent fusion partner in NSCLC.^57^, ^89^, ^91^ The second most common fusion partner in *RET*‐rearranged NSCLC is *CCDC6* (23%). Less frequently, fusion with *NCOA4* (2%), *EPHA5* (1%) and *PICALM* (1%) has been reported.^91^, ^92^ Notably, *RET*‐rearrangements are found in equal proportion in male and in female NSCLC patients. Among patients in the above‐mentioned global registry, a relatively large proportion, namely 63%, were never‐smokers, whilst 24% were former smokers and 10% reported to currently smoke. Nearly all the reported cases (98%) were adenocarcinomas. A total of 72% of these *RET*‐rearranged tumors were already at stage IV at the time of diagnosis. On the one hand, this suggests a relatively high metastatic potential, possibly caused by the *RET*‐fusion oncogene. On the other hand it could also mean that *RET*‐fusion occurs in later stages of carcinogenesis, or that the *RET* fusion‐oncogene is more readily found in patients with metastatic disease, since molecular testing is done in nonmetastatic cases less frequently.^46^, ^91^ Another example of oncogene addiction is NSCLC featuring aberrant expression of the proto‐oncogene *c‐MET* in addition to *EGFR* mutation.^93^ A study from 2018 revealed that cigarette smoking further augments oncogene addiction to *c‐MET* in NSCLC cells, suggesting that *MET*‐inhibition is a therapy option especially for lung cancer patients with a smoking history, or who are currently smoking. It is already known that cigarette smoking is associated with the insensitivity of NSCLC to treatment with *EGFR*‐TKIs. However, it is yet undetermined by which exact molecular mechanisms tobacco smoke affects *EGFR*‐TKI treatment. In this study about *c‐MET* in the context of NSCLC and tobacco smoke, it was shown that chronic exposure to cigarette smoke extract or tobacco smoke‐derived carcinogen benzo[α]pyrene, B[α]P, but not nicotine‐derived nitrosamine ketone (NNK), diminished treatment response to *EGFR*‐TKIs in wild‐type *EGFR*‐expressing NSCLC cells. Interestingly, TKI‐treatment did inhibit *EGFR* tyrosine kinase activity almost completely; however, no inhibitory effect on downstream Akt and ERK pathways was observed in B[α]P‐treated NSCLC cells.^93^ Cigarette smoke extract and B[α]P both transcriptionally upregulate the activity of the proto‐oncogene *c‐MET*, also activating its downstream Akt pathway, which could not be targeted by *EGFR*‐TKIs. Consecutively, the authors of this study silenced *c‐MET* which effectively reduced B[α]P‐induced activation of Akt. The cigarette smoke extract‐treated NSCLC cells were sensitive to treatment with the *c‐MET* inhibitor crizotinib. Thus, cigarette smoke evidently increases NSCLC‐oncogene addiction to *c‐MET*, but treatment with the *c‐MET* inhibitor crizotinib seems to be effective despite ongoing cigarette smoke exposure.^93^

### Circulating tumor DNA in association with mutational load

Circulating cell‐free tumor DNA fragments have been found in the bloodstream of patients suffering from malignant diseases since 2001.^94^ As cell‐free DNA also occurs in the bloodstream of healthy individuals, however to a much smaller proportion as compared to cancer patients, it is believed that cell‐free DNA derives from both tumor cells and nonmalignant cells.^95^, ^96^ It has also been reported that higher levels of circulating DNA are found when cells undergo apoptosis or necrosis.^94^ Recently, researchers have focused on cancer‐specific mutations found in the cell‐free DNA, which are also present in the primary tumor.^97^ Since the proportion of necrosis within rapidly proliferating and growing tumors increases, more cell free DNA is released. Generally, the more aggressive a tumor is growing, the more mutations are found, and therefore it is likely that mutated cell‐free tumor DNA increases with mutational load.^98^ In a study by Winther *et al*. levels of plasma mutant cell‐free DNA and metabolic tumor burden, measured by 18F‐fluoro‐d‐glucose positron emission tomography/computer tomography (PET/CT), were correlated. Additionally, the patients' survival times were correlated with the amount of mutated cell‐free DNA. Overall, 46 patients suffering from advanced NSCLC were included in the analysis. At the time of inclusion, blood was taken and PET/CT scans were performed. Cell‐free DNA was isolated and next‐generation sequencing was carried out by means of the AmpliSeq Colon and Lung Cancer Gene Panel V2. Metabolic tumor burden was determined volumetrically via the PET/CT. The authors of this interesting study report a significant correlation between the allele frequency of the most frequent mutations and metabolic tumor burden (*P* < 0.001).^98^ When mutated cell free DNA was detected, median overall survival was significantly shorter as compared to patients where no mutated cell‐free DNA was found (3.7 vs. 10.6 months, *P* < 0.019). Interestingly, this impact on survival was independent of the metabolic tumor burden, indicating that the mutational load is even more important for prediction of a patient's prognosis. Mutational load detected in circulating cell‐free DNA could become an important prognostic parameter in future everyday clinical practice.

A different study, conducted by Reynolds and colleagues, aimed at validating next generation sequencing technology for detecting gene mutations with residual cell pellets by means of liquid‐based cytology.^99^ *EGFR*‐mutation status in NSCLC samples is routinely outlined with allele‐specific polymerase chain reaction in liquid‐based cytology samples, for instance from endoscopic ultrasound‐guided fine needle aspiration. In this study, however, DNA extracted from liquid‐based cytology samples was tested with a multiplex‐amplified and enriched Ion AmpliSeq Cancer Hotspot sequencing panel, as well as the OneTouch 2 instrument. Consecutively, six NSCLC‐related genes covered by this sequencing panel (*BRAF*, *EGFR*, *ERBB2*, *KRAS*, *MET*, and phosphatidylinositol‐4,5‐bisphosphate 3‐kinase catalytic subunit a [*PIK3CA*]) were analyzed.^99^ Not surprisingly, the common *EGFR* sequence changes, including four *L858R* mutations, three exon 19 deletions and one exon 20 insertion, were found to correspond 100% between the assay platforms. Variants of the mutation hotspots *ERBB2*, *KRAS*, *MET*, and *PIK3CA* genes, commonly found in NSCLC, were also observed. Thus, next generation sequencing can obviously also be performed successfully in liquid‐based cytology samples of NSCLC.^99^

## Conclusion

Mutations frequently observed in NSCLC are of increasing relevance for choosing the most beneficial therapy regimen in a patient. Within the last few years, several new targeted therapies, specifically anchoring at NSCLC hotspot mutations, or at tumor‐specific fusion proteins, have been developed. The overall mutational load in a given tumor mass seems to have prognostic relevance, although data is inconclusive in case of oncogene‐addicted tumors that are primarily driven by a single mutation. Here, TMB might be associated with a better overall survival and PFS when immune therapy is administered. However, novel data from the CheckMate 227 and the Keynote 021 trial show differential results, suggesting that TMB might not be a reliable predictor of PFS, overall survival and response rate in NSCLC upon immunotherapy. A higher mutational burden is usually associated with an adverse outcome, since high TMB generally occurs in rapidly growing and advanced‐stage tumors. It has recently been demonstrated that the tumor microenvironment changes in accordance with overall mutational load, highlighting the connection between mutational status, total mutational load, aspects of the immunological microenvironment and, of course, treatment response and outcome.

## Disclosure

The authors of this manuscript declare that no relationship with private or public firms of any value has to be disclosed in the context of this article. No funding was received, and there are no financial or nonfinancial conflicts of interest.

## References

[1] Global Burden of Disease Cancer Collaboration , Fitzmaurice C , Akinyemiju TF *et al* Global, regional, and national cancer incidence, mortality, years of life lost, years lived with disability, and disability‐adjusted life‐years for 29 cancer groups, 1990 to 2016: A systematic analysis for the global burden of disease study. JAMA Oncol 2018; 4 (11): 1553–68. 29860482 10.1001/jamaoncol.2018.2706 PMC6248091

[2] Bray F , Ferlay J , Soerjomataram I , Siegel RL , Torre LA , Jemal A . Global cancer statistics 2018: GLOBOCAN estimates of incidence and mortality worldwide for 36 cancers in 185 countries. CA Cancer J Clin 2018; 68 (6): 394–424. 30207593 10.3322/caac.21492

[3] Siegel RL , Miller KD , Jemal A . Cancer statistics, 2018. CA Cancer J Clin 2018; 68 (1): 7–30. 29313949 10.3322/caac.21442

[4] Tallen G , Riabowol K . Keep‐ING balance: Tumor suppression by epigenetic regulation. FEBS Lett 2014; 588 (16): 2728–42. 24632289 10.1016/j.febslet.2014.03.011

[5] Ferlay J , Soerjomataram I , Ervik M *et al* GLOBOCAN 2012 v1.0, Cancer Incidence and Mortality Worldwide: IARC CancerBase No. 11. In: *International Agency for Research on Cancer*, *2013* Lyon, France; 2012.

[6] Wong MCS , Lao XQ , Ho K , Goggins WB , SLA T . Incidence and mortality of lung cancer: Global trends and association with socioeconomic status. Sci Rep 2017; 7: 14300. 29085026 10.1038/s41598-017-14513-7 PMC5662733

[7] Centers for Disease Control and Prevention . National Center for Health Statistics. *National Vital Statistics Report. Deaths: Vol. 61* Final Data for 2010. 2013.

[8] American Cancer Society . Cancer Facts & Figures 2014. American Cancer Society, Atlanta 2014.

[9] Woodard GA , Jones KD , Jablons DM . Lung cancer staging and prognosis. Cancer Treat Res 2016; 170: 47–75. 27535389 10.1007/978-3-319-40389-2_3

[10] Wakelee H , Kelly K , Edelman MJ . 50 years of progress in the systemic therapy of non‐small cell lung cancer. Am Soc Clin Oncol Educ Book 2014; 177–89. 10.14694/EdBook_AM.2014.34.177. 24857075 PMC5600272

[11] The American Cancer Society . *Immunotherapy for NSCLC* [Cited Jun 2019.] Available from URL: https://www.cancer.org/cancer/non-small-cell-lung-cancer/treating/immunotherapy.html

[12] Duruisseaux M , Rouquette I , Adam J *et al* Efficacy of PD‐1/PD‐L1 immune checkpoint inhibitors and PD‐L1 testing in thoracic cancers. Ann Pathol 2017; 37 (1): 61–78. 28162296 10.1016/j.annpat.2016.12.009

[13] Ksienski D , Wai ES , Croteau N *et al* Pembrolizumab for advanced nonsmall cell lung cancer: Efficacy and safety in everyday clinical practice. Lung Cancer 2019; 133: 110–6. 31200816 10.1016/j.lungcan.2019.05.005

[14] Sanford M , Scott LJ . Gefitinib: A review of its use in the treatment of locally advanced/metastatic non‐small cell lung cancer. Drugs 2009; 69 (16): 2303–28. 19852530 10.2165/10489100-000000000-00000

[15] Osmani L , Askin F , Gabrielson E , Li QK . Current WHO guidelines and the critical role of immunohistochemical markers in the subclassification of non‐small cell lung carcinoma (NSCLC): Moving from targeted therapy to immunotherapy. Semin Cancer Biol 2018; 52 (Pt 1): 103–9. 29183778 10.1016/j.semcancer.2017.11.019 PMC5970946

[16] Li N , Ou W , Ye X *et al* Pemetrexed‐carboplatin adjuvant chemotherapy with or without gefitinib in resected stage IIIA‐N2 non‐small cell lung cancer harbouring EGFR mutations: A randomized, phase II study. Ann Surg Oncol 2014; 21 (6): 2091–6. 24585406 10.1245/s10434-014-3586-9

[17] Lou Y , Dholaria B , Soyano A *et al* Survival trends among non‐small‐cell lung cancer patients over a decade: Impact of initial therapy at academic centers. Cancer Med 2018; 7 (10): 4932–42. 30175515 10.1002/cam4.1749 PMC6198232

[18] Offin M , Rizvi H , Tenet M *et al* Tumor mutation burden and efficacy of EGFR‐tyrosine kinase inhibitors in patients with EGFR‐mutant lung cancers. Clin Cancer Res 2019; 25 (3): 1063–9. 30045933 10.1158/1078-0432.CCR-18-1102 PMC6347551

[19] Cao D , Xu H , Xu X , Guo T , Ge W . High tumor mutation burden predicts better efficacy of immunotherapy: A pooled analysis of 103078 cancer patients. Oncoimmunology 2019; 8 (9): e1629258. 31428527 10.1080/2162402X.2019.1629258 PMC6685508

[20] Fox EJ , Salk JJ , Loeb LA . Exploring the implications of distinct mutational signatures and mutation rates in aging and cancer. Genome Med 2016; 8 (1): 30. 26987311 10.1186/s13073-016-0286-z PMC4797182

[21] Martincorena I , Campbell PJ . Somatic mutation in cancer and normal cells. Science 2015; 349 (6255): 1483–9. 26404825 10.1126/science.aab4082

[22] Risques RA , Kennedy SR . Aging and the rise of somatic cancer‐associated mutations in normal tissues. PLOS Genet 2018; 14 (1): e1007108. 29300727 10.1371/journal.pgen.1007108 PMC5754046

[23] Jia Q , Zhou J , Chen G *et al* Diversity index of mucosal resident T lymphocyte repertoire predicts clinical prognosis in gastric cancer. Oncoimmunology 2015; 4 (4): e1001230. 26137399 10.1080/2162402X.2014.1001230 PMC4485732

[24] Melendez B , Van Campenhout C , Rorive S , Remmelink M , Salmon I , D'Haene N . Methods of measurement for tumor mutational burden in tumor tissue. Transl Lung Cancer Res 2018; 7 (6): 661–7. 30505710 10.21037/tlcr.2018.08.02 PMC6249625

[25] McGranahan N , Furness AJ , Rosenthal R *et al* Clonal neoantigens elicit T cell immunoreactivity and sensitivity to immune checkpoint blockade. Science 2016; 351 (6280): 1463–9. 26940869 10.1126/science.aaf1490 PMC4984254

[26] Jia Q , Wu W , Wang Y *et al* Local mutational diversity drives intratumoral immune heterogeneity in non‐small cell lung cancer. Nat Commun 2018; 9 (1): 5361. 30560866 10.1038/s41467-018-07767-w PMC6299138

[27] Snyder A , Makarov V , Merghoub T *et al* Genetic basis for clinical response to CTLA‐4 blockade in melanoma. N Engl J Med 2014; 371 (23): 2189–99. 25409260 10.1056/NEJMoa1406498 PMC4315319

[28] Rizvi NA , Hellmann MD , Snyder A *et al* Cancer immunology. Mutational landscape determines sensitivity to PD‐1 blockade in non‐small cell lung cancer. Science 2015; 348 (6230): 124–8. 25765070 10.1126/science.aaa1348 PMC4993154

[29] Van Allen EM , Miao D , Schilling B *et al* Genomic correlates of response to CTLA‐4 blockade in metastatic melanoma. Science 2015; 350 (6257): 207–11. 26359337 10.1126/science.aad0095 PMC5054517

[30] Schumacher TN , Schreiber RD . Neoantigens in cancer immunotherapy. Science 2015; 348 (6230): 69–74. 25838375 10.1126/science.aaa4971

[31] Hellmann MD , Ciuleanu TE , Pluzanski A *et al* Nivolumab plus ipilimumab in lung cancer with a high tumor mutational burden. N Engl J Med 2018; 378 (22): 2093–104. 29658845 10.1056/NEJMoa1801946 PMC7193684

[32] Peters S . Abstract LBA7128 ‘Nivolumab (nivo) + low‐dose ipilimumab (ipi) vs platinum‐doublet chemotherapy (chemo) as first‐line (1L) treatment (tx) for advanced non‐small cell lung cancer (NSCLC): CheckMate‐227 part 1 final analysis’, Vol. 30 *Annals of Oncology*, *Congress Abstracts*, ESMO Congress, Barcelona 2019.

[33] Gandhi L , Rodriguez‐Abreu D , Gadgeel S *et al* Pembrolizumab plus chemotherapy in metastatic non‐small‐cell lung cancer. N Engl J Med 2018; 378 (22): 2078–92. 29658856 10.1056/NEJMoa1801005

[34] Garassino MC . KEYNOTE 189: Tumor Mutational Burden Not Significantly Associated with Efficacy of Pembrolizumab. *IASLC* 2019 World Conference on Lung Cancer, Barcelona 2019.

[35] Langer CJ , Gadgeel SM , Borghaei H *et al* Carboplatin and pemetrexed with or without pembrolizumab for advanced, non‐squamous non‐small‐cell lung cancer: A randomised, phase 2 cohort of the open‐label KEYNOTE‐021 study. Lancet Oncol 2016; 17 (11): 1497–508. 27745820 10.1016/S1470-2045(16)30498-3 PMC6886237

[36] Langer CJ . New KEYNOTE 021 Data Shows No Association with Tumor Mutational Burden. *IASLC* 2019 World Conference on Lung Cancer, Barcelona 2019.

[37] Vanderwalde A , Spetzler D , Xiao N , Gatalica Z , Marshall J . Microsatellite instability status determined by next‐generation sequencing and compared with PD‐L1 and tumor mutational burden in 11,348 patients. Cancer Med 2018; 7 (3): 746–56. 29436178 10.1002/cam4.1372 PMC5852359

[38] De Mello RA , Liu DJ , Aguiar PN , Tadokoro H . EGFR and EML4‐ALK updated therapies in non‐small cell lung cancer. Recent Pat Anticancer Drug Discov 2016; 11 (4): 393–400. 27491402 10.2174/1574892811666160803090944

[39] Rosell R , Carcereny E , Gervais R *et al* Erlotinib versus standard chemotherapy as first‐line treatment for European patients with advanced EGFR mutation‐positive non‐small‐cell lung cancer (EURTAC): A multicentre, open‐label, randomised phase 3 trial. Lancet Oncol 2012; 13 (3): 239–46. 22285168 10.1016/S1470-2045(11)70393-X

[40] Gupta R , Dastane AM , Forozan F *et al* Evaluation of EGFR abnormalities in patients with pulmonary adenocarcinoma: The need to test neoplasms with more than one method. Mod Pathol 2009; 22 (1): 128–33. 18997733 10.1038/modpathol.2008.182

[41] Shigematsu H , Gazdar AF . Somatic mutations of epidermal growth factor receptor signaling pathway in lung cancers. Int J Cancer 2006; 118 (2): 257–62. 16231326 10.1002/ijc.21496

[42] Suzuki M , Shigematsu H , Hiroshima K *et al* Epidermal growth factor receptor expression status in lung cancer correlates with its mutation. Hum Pathol 2005; 36 (10): 1127–34. 16226114 10.1016/j.humpath.2005.08.007

[43] Ladanyi M , Pao W . Lung adenocarcinoma: Guiding EGFR‐targeted therapy and beyond. Mod Pathol 2008; 21 (Suppl. 2): S16–22. 18437168 10.1038/modpathol.3801018

[44] Lee DH . Treatments for EGFR‐mutant non‐small cell lung cancer (NSCLC): The road to a success, paved with failures. Pharmacol Ther 2017; 174: 1–21. 28167215 10.1016/j.pharmthera.2017.02.001

[45] Liao BC , Lin CC , Lee JH , Yang JC . Optimal management of EGFR‐mutant non‐small cell lung cancer with disease progression on first‐line tyrosine kinase inhibitor therapy. Lung Cancer 2017; 110: 7–13. 28676222 10.1016/j.lungcan.2017.05.009

[46] Farago AF , Azzoli CG . Beyond ALK and ROS1: RET, NTRK, EGFR and BRAF gene rearrangements in non‐small cell lung cancer. Transl Lung Cancer Res 2017; 6 (5): 550–9. 29114471 10.21037/tlcr.2017.08.02 PMC5653525

[47] Gainor JF , Varghese AM , Ou SH *et al* ALK rearrangements are mutually exclusive with mutations in EGFR or KRAS: An analysis of 1,683 patients with non‐small cell lung cancer. Clin Cancer Res 2013; 19 (15): 4273–81. 23729361 10.1158/1078-0432.CCR-13-0318 PMC3874127

[48] Lin JJ , Ritterhouse LL , Ali SM *et al* ROS1 fusions rarely overlap with other oncogenic drivers in non‐small cell lung cancer. J Thorac Oncol 2017; 12 (5): 872–7. 28088512 10.1016/j.jtho.2017.01.004 PMC5403618

[49] Shaw AT , Ou SH , Bang YJ *et al* Crizotinib in ROS1‐rearranged non‐small‐cell lung cancer. N Engl J Med 2014; 371 (21): 1963–71. 25264305 10.1056/NEJMoa1406766 PMC4264527

[50] Shaw AT , Kim DW , Nakagawa K *et al* Crizotinib versus chemotherapy in advanced ALK‐positive lung cancer. N Engl J Med 2013; 368 (25): 2385–94. 23724913 10.1056/NEJMoa1214886

[51] Shaw AT , Felip E , Bauer TM *et al* Lorlatinib in non‐small‐cell lung cancer with ALK or ROS1 rearrangement: An international, multicentre, open‐label, single‐arm first‐in‐man phase 1 trial. Lancet Oncol 2017; 18 (12): 1590–9. 29074098 10.1016/S1470-2045(17)30680-0 PMC5777233

[52] Drilon A , Bergagnini I , Delasos L *et al* Clinical outcomes with pemetrexed‐based systemic therapies in RET‐rearranged lung cancers. Ann Oncol 2016; 27 (7): 1286–91. 27056998 10.1093/annonc/mdw163 PMC4922319

[53] Ferrara R , Auger N , Auclin E , Besse B . Clinical and translational implications of RET rearrangements in non‐small cell lung cancer. J Thorac Oncol 2018; 13 (1): 27–45. 29128428 10.1016/j.jtho.2017.10.021

[54] Baik CS , Myall NJ , Wakelee HA . Targeting BRAF‐mutant non‐small cell lung cancer: From molecular profiling to rationally designed therapy. Oncologist 2017; 22 (7): 786–96. 28487464 10.1634/theoncologist.2016-0458 PMC5507646

[55] Planchard D , Besse B , Groen HJM *et al* Dabrafenib plus trametinib in patients with previously treated BRAF(V600E)‐mutant metastatic non‐small cell lung cancer: An open‐label, multicentre phase 2 trial. Lancet Oncol 2016; 17 (7): 984–93. 27283860 10.1016/S1470-2045(16)30146-2 PMC4993103

[56] Rolfo C , Ruiz R , Giovannetti E *et al* Entrectinib: A potent new TRK, ROS1, and ALK inhibitor. Expert Opin Investig Drugs 2015; 24 (11): 1493–500. 10.1517/13543784.2015.1096344 26457764

[57] Takeuchi K , Soda M , Togashi Y *et al* RET, ROS1 and ALK fusions in lung cancer. Nat Med 2012; 18 (3): 378–81. 22327623 10.1038/nm.2658

[58] Soda M , Choi YL , Enomoto M *et al* Identification of the transforming EML4‐ALK fusion gene in non‐small‐cell lung cancer. Nature 2007; 448 (7153): 561–6. 17625570 10.1038/nature05945

[59] Gainor JF , Shaw AT . Novel targets in non‐small cell lung cancer: ROS1 and RET fusions. Oncologist 2013; 18 (7): 865–75. 23814043 10.1634/theoncologist.2013-0095 PMC3720641

[60] Cancer Genome Atlas Research Network . Comprehensive molecular profiling of lung adenocarcinoma. Nature 2014; 511 (7511): 543–50. 25079552 10.1038/nature13385 PMC4231481

[61] Ahn HK , Jeon K , Yoo H *et al* Successful treatment with crizotinib in mechanically ventilated patients with ALK positive non‐small‐cell lung cancer. J Thorac Oncol 2013; 8 (2): 250–3. 23328551 10.1097/JTO.0b013e3182746772

[62] Califano R , Greystoke A , Lal R , Thompson J , Popat S . Management of ceritinib therapy and adverse events in patients with ALK‐rearranged non‐small cell lung cancer. Lung Cancer 2017; 111: 51–8. 28838397 10.1016/j.lungcan.2017.06.004

[63] Peters S , Camidge DR , Shaw AT *et al* Alectinib versus Crizotinib in untreated ALK‐positive non‐small‐cell lung cancer. N Engl J Med 2017; 377 (9): 829–38. 28586279 10.1056/NEJMoa1704795

[64] Solomon BJ , Mok T , Kim DW *et al* First‐line crizotinib versus chemotherapy in ALK‐positive lung cancer. N Engl J Med 2014; 371 (23): 2167–77. 25470694 10.1056/NEJMoa1408440

[65] Barlesi F , Mazieres J , Merlio JP *et al* Routine molecular profiling of patients with advanced non‐small‐cell lung cancer: Results of a 1‐year nationwide programme of the French cooperative thoracic intergroup (IFCT). Lancet 2016; 387 (10026): 1415–26. 26777916 10.1016/S0140-6736(16)00004-0

[66] Hofman P . ALK in non‐small cell lung cancer (NSCLC) pathobiology, epidemiology, detection from tumor tissue and algorithm diagnosis in a daily practice. Cancers (Basel) 2017; 9 (8). 10.3390/cancers9080107. PMC5575610 28805682

[67] Le T , Gerber DE . ALK alterations and inhibition in lung cancer. Semin Cancer Biol 2017; 42: 81–8. 27637426 10.1016/j.semcancer.2016.08.007 PMC5316306

[68] Liu J , Cui S , Pan F *et al* Feasibility of continuing crizotinib therapy after RECIST‐PD in advanced non‐small cell lung cancer patients with ALK/ROS‐1 mutations. J Cancer 2018; 9 (10): 1863–9. 29805713 10.7150/jca.24950 PMC5968775

[69] Gadgeel SM . Sequencing of ALK inhibitors in ALK+ non‐small cell lung cancer. Curr Treat Options Oncol 2017; 18 (6): 36. 28534251 10.1007/s11864-017-0479-8

[70] Camidge DR , Bang YJ , Kwak EL *et al* Activity and safety of crizotinib in patients with ALK‐positive non‐small‐cell lung cancer: Updated results from a phase 1 study. Lancet Oncol 2012; 13 (10): 1011–9. 22954507 10.1016/S1470-2045(12)70344-3 PMC3936578

[71] Metro G , Tazza M , Matocci R , Chiari R , Crino L . Optimal management of ALK‐positive NSCLC progressing on crizotinib. Lung Cancer 2017; 106: 58–66. 28285695 10.1016/j.lungcan.2017.02.003

[72] Veldore VH , Patil S , Satheesh CT *et al* Genomic profiling in a homogeneous molecular subtype of non‐small cell lung cancer: An effort to explore new drug targets. Indian J Cancer 2015; 52 (2): 243–8. 26853422 10.4103/0019-509X.175843

[73] Choughule A , Noronha V , Joshi A *et al* Epidermal growth factor receptor mutation subtypes and geographical distribution among Indian non‐small cell lung cancer patients. Indian J Cancer 2013; 50 (2): 107–11. 23979200 10.4103/0019-509X.117023 PMC5808828

[74] Mehta J . Molecular epidemiology of epidermal growth factor receptor mutations in lung cancers in Indian population. Indian J Cancer 2013; 50 (2): 102–6. 23979199 10.4103/0019-509X.117019

[75] Brabender J , Danenberg KD , Metzger R *et al* Epidermal growth factor receptor and HER2‐neu mRNA expression in non‐small cell lung cancer is correlated with survival. Clin Cancer Res 2001; 7 (7): 1850–5. 11448895

[76] Hirsch FR , Scagliotti GV , Langer CJ , Varella‐Garcia M , Franklin WA . Epidermal growth factor family of receptors in preneoplasia and lung cancer: Perspectives for targeted therapies. Lung Cancer 2003; 41 (Suppl. 1): S29–42. 12867060 10.1016/s0169-5002(03)00137-5

[77] Marchetti A , Martella C , Felicioni L *et al* EGFR mutations in non‐small‐cell lung cancer: Analysis of a large series of cases and development of a rapid and sensitive method for diagnostic screening with potential implications on pharmacologic treatment. J Clin Oncol 2005; 23 (4): 857–65. 15681531 10.1200/JCO.2005.08.043

[78] Rusch V , Klimstra D , Venkatraman E , Pisters PW , Langenfeld J , Dmitrovsky E . Overexpression of the epidermal growth factor receptor and its ligand transforming growth factor alpha is frequent in resectable non‐small cell lung cancer but does not predict tumor progression. Clin Cancer Res 1997; 3 (4): 515–22. 9815714

[79] Bylicki O , Barazzutti H , Paleiron N , Margery J , Assie JB , Chouaid C . First‐line treatment of non‐small‐cell lung cancer (NSCLC) with immune checkpoint inhibitors. BioDrugs 2019; 33 (2): 159–71. 30825132 10.1007/s40259-019-00339-4

[80] Gou LY , Wu YL . Prevalence of driver mutations in non‐small‐cell lung cancers in the People's Republic of China. Lung Cancer (Auckl) 2014; 5: 1–9. 28210137 10.2147/LCTT.S40817 PMC5217505

[81] Herbst RS , Morgensztern D , Boshoff C . The biology and management of non‐small cell lung cancer. Nature 2018; 553 (7689): 446–54. 29364287 10.1038/nature25183

[82] Metro G , Chiari R , Bennati C *et al* Clinical outcome with platinum‐based chemotherapy in patients with advanced nonsquamous EGFR wild‐type non‐small‐cell lung cancer segregated according to KRAS mutation status. Clin Lung Cancer 2014; 15 (1): 86–92. 24139827 10.1016/j.cllc.2013.08.002

[83] Zhao N , Zhang XC , Yan HH , Yang JJ , Wu YL . Efficacy of epidermal growth factor receptor inhibitors versus chemotherapy as second‐line treatment in advanced non‐small‐cell lung cancer with wild‐type EGFR: A meta‐analysis of randomized controlled clinical trials. Lung Cancer 2014; 85 (1): 66–73. 24780111 10.1016/j.lungcan.2014.03.026

[84] Facchinetti F , Rossi G , Bria E *et al* Oncogene addiction in non‐small cell lung cancer: Focus on ROS1 inhibition. Cancer Treat Rev 2017; 55: 83–95. 28342334 10.1016/j.ctrv.2017.02.010

[85] Dai Y , Wei Q , Schwager C *et al* Oncogene addiction and radiation oncology: Effect of radiotherapy with photons and carbon ions in ALK‐EML4 translocated NSCLC. Radiat Oncol 2018; 13 (1): 1. 29304828 10.1186/s13014-017-0947-0 PMC5756447

[86] Schuchardt A , D'Agati V , Larsson‐Blomberg L , Costantini F , Pachnis V . Defects in the kidney and enteric nervous system of mice lacking the tyrosine kinase receptor ret. Nature 1994; 367 (6461): 380–3. 8114940 10.1038/367380a0

[87] Nakamura T , Ishizaka Y , Nagao M , Hara M , Ishikawa T . Expression of the ret proto‐oncogene product in human normal and neoplastic tissues of neural crest origin. J Pathol 1994; 172 (3): 255–60. 8195928 10.1002/path.1711720305

[88] Santoro M , Carlomagno F , Hay ID *et al* Ret oncogene activation in human thyroid neoplasms is restricted to the papillary cancer subtype. J Clin Invest 1992; 89 (5): 1517–22. 1569189 10.1172/JCI115743 PMC443023

[89] Kohno T , Ichikawa H , Totoki Y *et al* KIF5B‐RET fusions in lung adenocarcinoma. Nat Med 2012; 18 (3): 375–7. 22327624 10.1038/nm.2644 PMC6430196

[90] Wang R , Hu H , Pan Y *et al* RET fusions define a unique molecular and clinicopathologic subtype of non‐small‐cell lung cancer. J Clin Oncol 2012; 30 (35): 4352–9. 23150706 10.1200/JCO.2012.44.1477

[91] Gautschi O , Milia J , Filleron T *et al* Targeting RET in patients with RET‐rearranged lung cancers: Results from the global, multicenter RET registry. J Clin Oncol 2017; 35 (13): 1403–10. 28447912 10.1200/JCO.2016.70.9352 PMC5559893

[92] Drilon A , Wang L , Hasanovic A *et al* Response to Cabozantinib in patients with RET fusion‐positive lung adenocarcinomas. Cancer Discov 2013; 3 (6): 630–5. 23533264 10.1158/2159-8290.CD-13-0035 PMC4160032

[93] Tu CY , Cheng FJ , Chen CM *et al* Cigarette smoke enhances oncogene addiction to c‐MET and desensitizes EGFR‐expressing non‐small cell lung cancer to EGFR TKIs. Mol Oncol 2018; 12 (5): 705–23. 29570930 10.1002/1878-0261.12193 PMC5928373

[94] Jahr S , Hentze H , Englisch S *et al* DNA fragments in the blood plasma of cancer patients: Quantitations and evidence for their origin from apoptotic and necrotic cells. Cancer Res 2001; 61 (4): 1659–65. 11245480

[95] Mouliere F , Thierry AR . The importance of examining the proportion of circulating DNA originating from tumor, microenvironment and normal cells in colorectal cancer patients. Expert Opin Biol Ther 2012; 12 (Suppl. 1): S209–15. 22594497 10.1517/14712598.2012.688023

[96] Thierry AR , El Messaoudi S , Gahan PB , Anker P , Stroun M . Origins, structures, and functions of circulating DNA in oncology. Cancer Metastasis Rev 2016; 35 (3): 347–76. 27392603 10.1007/s10555-016-9629-x PMC5035665

[97] Wan JCM , Massie C , Garcia‐Corbacho J *et al* Liquid biopsies come of age: Towards implementation of circulating tumour DNA. Nat Rev Cancer 2017; 17 (4): 223–38. 28233803 10.1038/nrc.2017.7

[98] Winther‐Larsen A , Demuth C , Fledelius J *et al* Correlation between circulating mutant DNA and metabolic tumour burden in advanced non‐small cell lung cancer patients. Br J Cancer 2017; 117 (5): 704–9. 28683468 10.1038/bjc.2017.215 PMC5572172

[99] Reynolds JP , Zhou Y , Jakubowski MA *et al* Next‐generation sequencing of liquid‐based cytology non‐small cell lung cancer samples. Cancer Cytopathol 2017; 125 (3): 178–87. 28085233 10.1002/cncy.21812

